# Supplementary material for: Prevalence and Characteristics of Violence against Paramedics in a Single Canadian Site
Source: Int J Environ Res Public Health. 2023 Aug 24;20(17):6644. doi: 10.3390/ijerph20176644 (PMC10487157; doi:10.3390/ijerph20176644)
Supplement: Supplementary file 1 [file ijerph-20-06644-s001.zip › ijerph-2531816-supplementary.pdf]

mDOCS

External Violence Incident Report

T- T+

Save As Draft and Close | Submit and Close

Reporting Section

To Whom Does This Form Apply: -- Select Option --	Call Number:	Incident Date/Time:	Incident Type: ? Select All That Apply -- Select Option(s) --

Identify Source: Select All That Apply -- Select Option(s) --	Please specify other source:	Circumstances: Select All That Apply -- Select Option(s) --
Location of Incident: Select All That Apply -- Select all that apply --	At this time are you emotionally impacted as a result of the incident: Yes / No / I'm Uncertain	Were you physically harmed as a result of the incident: Yes / No

What happened? Please be specific and detailed.

Due to privacy concerns please do not include any of your own personal Medical Information

Was a hazard flag communicated by CACC: Yes / No	Do you recommend the creation of a hazard flag for violent behavior: Yes / No	Confirm criteria is met for creation of a hazard flag: ? Yes / No

Did police attend the call: Yes / No	Did you request Police? : Yes / No / N/A	Did you activate a 10-2000 : Yes / No / N/A	Police badge number(s) :	Was Police Response Helpful: Yes / No	Describe what they did:

Was a Superintendent involved at any time during or following call? -- Select Option(s) --	Would you like a Superintendent to follow up with you about this incident: Yes / No

Call Specifications

Service Name: -- Select Option --	Call Date:

Vehicle Number: -- Select Option --	UTM Code:	Dispatch Priority: -- Select Option --	Return Priority: -- Select Option --
Pickup Location:	Patient's Name:	Patient's Address:	Chief Complaint:

Dispatch Problem Code: -- Select Option --	Primary Problem Code: -- Select Option --	Interventions: -- Select Option(s) --
Crew List: -- Select Option(s) --	Report Creator: -- Select Option --	Report Creator Role: -- Select Option --

Call Received:	Crew Notified:	Crew Mobile:	Arrive Scene:	Depart Scene:

Arrive Destination:	Date Report Completed:	Depart Destination:	Arrive Base:

Opt-Out of Research

☐ I do not want this form used for research purposes ?

Superintendent Review Section - Management Use Only

☐ Significant Incident ?

Incident Entered in RAIDR: Yes / No	Was a hazard flag created or extended for this call location: For guidance, please refer to OHSA section 32.0.5 ? Yes / No	If NO new hazard flag created or extended, why not? Non-Residential / Flag Already Exists / Other	Please specify reason for not creating/extending hazard flag:

Notes

Ex. If Paramedic requested Superintendent follow up, please detail follow up plan.

Superintendent: -- Select Option --	Superintendent will follow up: Yes / No	☐ Follow Up/Completion pending:

Save As Draft and Close | Submit and Close

Version Number **2020.10.05**
Revision Number **2022.06.24**
Form ID **9829B5CC-E773-4523-A9AB-A081D70E6BCA**
Interdev Technologies Inc. —
